# A New Routine for Analyzing Brief Symptom Inventory Profiles in Chronic Pain Patients to Evaluate Psychological Comorbidity

**DOI:** 10.3389/fpsyg.2021.692545

**Published:** 2021-09-30

**Authors:** Gabriele Helga Franke, Melanie Jagla-Franke, Dieter Küch, Katja Petrowski

**Affiliations:** ^1^Division of Psychology of Rehabilitation, University of Applied Sciences Magdeburg and Stendal, Stendal, Germany; ^2^Klinik Hochstaufen, Reha-Zentrum Bayerisch Gmain, Bayerisch Gmain, Germany; ^3^Division of Medical Psychology and Medical Sociology, Department of Psychosomatic Medicine and Psychotherapy, University Medical Center of the Johannes Gutenberg University of Mainz, Mainz, Germany; ^4^Department of Internal Medicine III, Dresden University of Technology, Dresden, Germany

**Keywords:** psychological distress, BSI, new case definition, four groups, orthopedic patients

## Abstract

**Question:** Comorbidity, i.e., additional psychological distress in patients already suffering from chronic somatic diseases (e.g., orthopedic conditions) is of growing importance. The quality of analyzing and interpreting the often used Brief Symptom Inventory (BSI) used with orthopedic patients should improve by employing a new “case definition” of four groups (instead of two) of differentially psychologically distressed patients instead of two groups as before.

**Methods:** Four groups with the different psychological distress definitions of “no,” “mild,” “remarkable,” and “severe” were to be analyzed from a group of 639 orthopedic patients in inpatient rehabilitation clinics. The BSI is transformed into T values (*M*=50, SD=10). There is “no” distress if no T [two scales] is ≥60 and “mild” distress if T [two scales] and/or T [GSI] is ≥60 and <63. If T [two scales] and/or T [GSI] is ≥63 and <70, it is “remarkable,” and if T [two scales] and/or T [GSI] ≥70, it speaks for “severe” psychological distress.

**Results:** The new tool for analyzing psychological distress based on the T-scores of the BSI resulted in the following four groups: No psychological distress (41.9%): unspecific health-related information stands for a useful intervention. About 13.3% demonstrated low psychological distress: shorter diagnostic interviews and a few more diagnostic examinations led to a low-level outpatient group program to improve health and well-being in a preventive sense; one repeated measurement in 4weeks is advised. Remarkable psychological distress (26%): in-depth exploration using interviews, tests, and questionnaires to choose specific interventions in a single and/or group setting, outpatient or inpatient treatment; repeated measurements and process control. About 18.8% reported severe psychological distress: in-depth exploration led to specific interventions in a single and/or group setting, almost an inpatient setting; immediately crisis intervention and high-frequent process control.

**Conclusion:** The new evaluation strategy of the BSI should improve practice and research; further investigation is necessary.

## Introduction

Chronic or persistent pain (≥3months) is a relatively common experience in adults and a well-known cause of job-related disability and absenteeism (Breivik et al., 2006). The high prevalence of persistent pain is linked to an equally high prevalence of comorbid psychological distress (Rice et al., 2016). Joint disorders, such as arthritis, are recognized as the most common cause of chronic joint pain (ib.). Persistent pain contributes to an array of adverse consequences that include psychological distress, interference with physical and role functioning and instrumental activities of daily living, and ordinary tasks. Persistent pain may be considered as one of the most pervasive and expensive health care problems in the twenty-first century due to the high prevalence of adverse responses among the individuals affected (Breivik et al., 2006; Rice et al., 2016).

The psychological comorbidity – easily defined as additional psychological distress in chronic somatically ill patients – is of importance, because these patients report “a substantially reduced psychosocial and physical quality of life” (Baumeister et al., 2011, p. 275). About 25% of all medically ill patients in behavioral medicine and somatic rehabilitation hospitals suffer from serious mental health problems. Due to the close association of physical diseases and mental disorders, early and precise detection of psychological distress is of high clinical importance (Härter et al., 2004).

A very early study (Jamison et al., 1988) supported the usefulness of the SCL-90 (Derogatis, 1977) to analyze the psychological distress in pain patients in rehabilitative settings. An investigation of 4.496 cancer patients with the Brief Symptom Inventory (BSI; Derogatis and Melisaratos, 1983), a short version of SCL-90, showed an overall prevalence rate of distress of 35.1% (Zabora et al., 2001). The “case definitions” of SCL-90 and BSI were used: positive cases can be identified by a Global Severity Index (GSI) score of T≥63 or any two subscales where the T-score is ≥63 (Derogatis and Melisaratos, 1983; Franke, 2014, 2017). Therefore, the BSI can be used to evaluate psychological distress as “The BSI possesses characteristics that are more suitable for screening than other instruments such as the General Health Questionnaire or the Hospital Anxiety and Depression Scale” (Zabora et al., 2001, p. 21–22). In line with this, the Brief Symptom Checklist (BSCL) is often used in the evaluation of psychological distress in psychotherapeutic inpatients and outpatients (Busmann et al., 2019) as well as of students (Franke et al., 2017).

Furthermore, several studies investigating the psychological distress of (chronic) pain patients used the BSI (Carson et al., 2006) or the BSCL (Küch et al., 2016; Franke, 2017). In summary, the SCL-90 and the BSI are common tools used in rehabilitation settings; in Germany, approximately every second psychologist working in rehabilitation settings uses them (Franke et al., 2019).

However, there are two aspects that have increasingly come into focus when using the case definition in SCL procedures: on the one hand, the consideration of psychological distress which is still below the usual threshold but already measurable thus requiring psychological intervention (Hadlaczky et al., 2014). This calls for measures of prevention. On the other hand, there is the need to offer direct and immediate intervention and/or crisis intervention to particularly distressed patients (Walker et al., 2015). Therefore, a new evaluation strategy for the SCL-90 and the BSI should lead to a differentiated assessment of comorbidity.

The aim of the present study was to investigate a new routine for analyzing Brief Symptom Inventory profiles in chronic pain patients, since it is important to recognize and treat psychological distress as comorbidity in these medically ill patients (Baumeister et al., 2011). For this purpose, patients of orthopedic rehabilitation facilities were chosen, as 25% of medically ill patients suffer from serious mental health problems with a high risk of chronification. In these orthopedic rehab samples, osteoarthritis diseases were present as an underlying cause with the highest comorbidity of psychological distress (31%, Härter et al., 2004). In addition, patients with osteoporosis and diabetic foot syndrome showed a high comorbidity with anxiety symptoms and depression as well (Atteritano et al., 2013; Fernandes et al., 2016). In this patient collective, the placement into the most suitable treatment is of the highest relevance in order to avoid chronification, development of further comorbidities, postoperative complications, and an increase in mortality risk (Naicker et al., 2017; Pscherer et al., 2017). Therefore, a new routine for analyzing Brief Symptom Inventory profiles in chronic pain patients is of the greatest importance for identifying risk patients and placing them in the most suitable treatment facility for psychological distress.

## Materials and Methods

This new routine to analyze the BSCL will be reviewed in a sample of 639 orthopedic patients who underwent rehabilitation in the Paracelsus-Clinic an der Gande, Bad Gandersheim, Germany, between 2012 and 2016 (Franke, 2014). The mean age of the sample was 52.77 (±8.8); 147 (23%) were male and 492 (77%) were female; 239 (59.5%) were married and 163 (40.5%) were not married; and 366 (91%) were employed and 36 (9%) were unemployed. The most common diagnosis in rehabilitation facilities are osteoarthritis diseases, especially of the knee and hip joints. These diseases account for 46% of all musculoskeletal cases in rehabilitation facilities. Various deformities and diseases of the spine and back, respectively, are the second most common reason for rehabilitation stays, accounting for about 41% of all cases. These include back pain (88.147 cases), disc damage (53.944 cases), and spondylopathies (wear-related changes in the vertebral bodies, 48.834 cases). Other diagnoses include joint inflammation, muscle diseases, or osteoporosis (see also Database of the Federal Statistical Office of Germany, 2021).

However, further information on patients in rehabilitation facilities, such as to the patients’ comorbidities, were not available due to the anonymization of the patients’ data. In medical rehabilitation, comorbidity is primarily examined as psychological stress. With the help of an extensive study of 1,750 patients in orthopedic or CHD rehabilitation, Härter et al. (2004) proved that 31% of the patients in orthopedic rehabilitation and 20% of the patients in CHD rehabilitation had a psychological illness (comorbidity). If patients have another chronic disease in the foreground, such as diabetes mellitus, they are assigned to rehabilitation for digestive and metabolic diseases (Schmidt et al., 2018). However, one intersection between diabetes mellitus and orthopedic rehabilitation is diabetic foot syndrome as well as diabetic foot ulcer, which a small group of diabetics may develop and may lead to amputation. This rather rare clinical picture of amputation (Greitemann, 2009) was not represented in our collective.

### Measures

Brief Symptom Inventory (Franke, 2017): This self-report inventory assesses psychopathology and psychological distress; it requires only 10min to complete. By recalling the 7days prior to the date of the test, the BSI items are judged on a five-point Likert scale ranging from “not at all” (0) to “extremely” (4).

The 53 items measure the nine dimensions: somatization (SOM), obsessive-compulsive (O-C), interpersonal sensitivity (I-S), depression (DEP), anxiety (ANX), anger-hostility (HOS), phobic anxiety (PHOB), paranoid ideation (PAR), and psychoticism (PSY) by computing sum scores and divide them by the number of items performing the scale. Three summary scores are computed regarding the 53 items: the GSI is the well-known global score (summing all 53 responses and dividing them by 53); the Positive Symptom Total (PST) counts the items with an answer >0 and the Positive Symptom Distress Index (PSDI) that means summing up the 53 items and dividing them by the PST. T-scores are computed on the basis of representative data divided by age and gender (Franke, 2017).

Reliability in a sample of 402 pain patients ranged from Cronbach’s Alpha=0.74 (HOS) to Alpha=0.90 (DEP) regarding the scales; the reliability of the Global Score GSI was Alpha=0.97 (Franke, 2017). Computing the well-known case definition in the pain sample resulting in the two groups of remarkably distressed and not distressed patients, we found 239 (59.5%) not distressed and 163 (40.5%) distressed patients (Franke, 2017).

Patient Health Questionnaire (PHQ-4, Kroenke et al., 2009): The four-item PHQ measure of anxiety and depressive symptoms has been demonstrated as a general marker for psychological distress (Kroenke et al., 2009). The PHQ-4 was used to investigate a large cohort of senior citizens suffering from back pain (Jarvik et al., 2014).

Ultra-Short-Screening (Küch et al., 2013): Two items regarding pain and two single items regarding family as well as vocational problems were answered using a four-point Likert scale from 0–not at all, 1–on single days, 2–more than half of the days, 3–nearly every day, and 4–at least every day. These questions are often used in rehabilitation settings (Küch et al., 2013); together with the PHQ data, the results are used for step-by-step diagnostic procedures (Schmidt et al., 2019) to monitor interventions in rehabilitation (Schmidt et al., 2019).

### New Groups of Case Definition

The case definition postulated by Derogatis and Melisaratos (1983) adopted for the German manuals (Franke, 2014, 2017) is T [GSI] or T [two scales] ≥63; this leads to two groups, namely subjects with and subjects without psychological distress. The impetus for the new case definition, leading to four groups, was theoretically driven by psychometric aspects. Based on the representative sample (Franke, 2017), mean (*T*=50) plus one SD (*T*=10) leads to the cut-off *T*=60. Based on the ground of the original cut-off (Derogatis and Melisaratos, 1983; Franke, 2017), *T*=63 is used as a threshold, and *T*=70 is used as a cut-off for psychometric reasons (mean plus two SDs). This leads to the point that prevention and higher distress can be viewed in a more differentiated manner.

(1) Prevention: If only those with at least two scales and/ or the T [GSI] ≥63 are recorded as mentally distressed, it appears that slight psychological distress is overlooked. This might also lead to an underestimation of the psychological stress in groups and in individuals from the fact that the existing and measurable psychological stress between *T*≥60 and *T*<63 is overlooked. As a result, low-threshold interventions for psychological stabilization may not take place, and the burden may increase even though the individual is in contact with the help system. By resorting to psychometrics (mean plus one SD=50+10=60), a new threshold is introduced which leads to the recognition of this group of lightly stressed patients. (2) High(er) distress: The group of the psychologically distressed subjects appears too undifferentiated. Within the group of the psychologically distressed, there should be another psychometrically justifiable threshold in order to separate the remarkably distressed from the severely distressed. The psychometric threshold “mean value plus two SDs” (*M*+2 SD=50+20=70) is recommended. Individuals with at least two scales and/or TGSI≥70 show strong to very strong distress and should be treated with priority in relation to the intervention. (3) At the same time, the known case definition should be retained. The four new groups can therefore be combined into group 1 and 2 (old group “no case”) and 3 and 4 (old group “case”) and at the same time differentiated into “light exposure–group 2” and “severe exposure–group 4.” The new group 1 can thus be regarded as not burdened, and group 3 as remarkable but not difficult.

The new case definition (Franke, 2020; Franke et al., 2021; see Table 1) differentiates between four groups:

“no” distress: T [two scales]≥60.
“mild” distress: T [two scales] and/or T [GSI]≥60 and <63.
“remarkable” distress: T [two scales] and/or T [GSI]≥63 and <70.
“severe” distress: T [two scales] and/or T [GSI]≥70.

**Table 1:** New case definition for Brief Symptom Inventory (BSI) to evaluate psychological distress: the four groups according to the new case definition as well as recommendations for further diagnostics and interventions are shown.

	Psychological distress			
	No	Mild	Remarkable	Severe
Definition	No T [two scales]≥60	T [two scales] and/or T [GSI]≥60 and <63	T [two scales] and/or T [GSI]≥63 and <70	T [two scales] and/or T [GSI]≥70
Next diagnostic steps				
1. Retesting	When needed	In 4weeks	Frequent examination	Frequent examination
2. Interview	When needed	Short diagnostic interview	Diagnostic interview	Diagnostic interview
3. Further testing	If T [scale]≥60, then short exploration of the items of the scale	Exploration of the scales T [scale]≥60 and <63 and deepening through few more tests	Exploration of the scales T [scale]≥63 and <70 and deepening through more tests	Exploration of the scales T [scale]≥70 and deepening through more tests
Possible interventions	Health-promoting information	Health-promoting group-interventions, low threshold, outpatient	Specific individual and/or group programs, outpatients, optionally inpatient	Specific individual and/or group programs, predominantly inpatient
Levels of interventions	Information	Prevention	Intervention	Urgent or crisis intervention

## Results

### New Case Definition

In a re-analysis of a sample of 639 patients undergoing inpatient orthopedic rehabilitation, the classic case definition at BSI resulted in 44.8% of psychologically stressed patients. The new case definition differentiates as follows (see Table 2):

- No psychological distress was found in 268 (41.9%) patients. Here, the T-value for the global characteristic value GSI was *T*=47 (± 8); all T-scores ranged below *T*=60.
- Eighty-five (13.3%) patients showed mild psychological distress and should be accompanied more attentively [T (GSI)=57±3].
- Remarkable psychological distress was found in 166 (26%) patients [T (GSI)=63±3], and the scale scores ranged between the minimum T [PHOB]=58 (±9) and the maximum T [O-C]=62 (±6). Six more T-scores were ≥60: T [SOM]=61 (±8), T [I-S]=61 (±6), T [ANX]=61 (±6), T [DEP]=60 (±6), T [HOS]=60 (±7), and T [PAR]=60 (±7).
- One hundred and twenty (18.8%) of the orthopedic patients were severely psychologically distressed [T (GSI)=71±5], and the scale values were between the minimum T [PHOB]=66 (±8) and the maximum T [O-C]=71 (±5). The T-scores of the remaining seven scales were: T [I-S]=70 (±7), T [DEP]=69 (±6), T [ANX]=69 (±7), T [PAR]=69 (±8), T [SOM]=68 (±7), T [HOS]=68 (±9), and T [PSY]=67 (±8).

**Table 2:** BSI profile of the four different psychologically distressed groups (T-scores; mean and standard deviation).

Psychological distress	No	Mild	Remarkable	Severe	Sum	Reliability (Cronbach’s Alpha)
	No two T [scales] ≥60	Two or more T [scales] and/or T [GSI]≥60 and <63	Two or more T [scales] and/or T [GSI]≥63 and <70	Two or more T [scales] and/or T [GSI]≥70		
	268 (41.9%)	85 (13.3%)	166 (26%)	120 (18.8%)	639	
SOM	50.75±7.98	57.37±6.51	61.10±8.06	68.02±6.82	57.56±10.05	*α* =0.81
O-C	47.63±7.63	55.81±6.77	62.25±6.03	70.75±4.91	56.86±11.14	*α* =0.89
I-S	46.32±5.45	54.95±6.94	60.83±6.07	69.78±6.78	55.64±10.86	*α* =0.84
DEP	48.00±5.86	54.20±6.92	60.13±5.58	68.74±5.56	55.87±9.84	*α* =0.89
ANX	47.22±6.88	56.37±6.62	60.72±6.33	68.86±7.26	56.01±10.73	*α* =0.85
HOS	45.54±6.26	53.49±7.35	59.90±6.84	68.32±8.71	54.61±11.27	*α* =0.73
PHOB	47.40±4.60	53.44±6.83	57.90±8.47	65.78±8.21	54.38±9.72	*α* =0.79
PAR	46.35±6.35	54.14±7.05	59.96±6.74	68.80±7.93	55.14±10.99	*α* =0.83
PSY	46.94±4.64	53.81±6.56	58.75±7.33	67.39±7.87	54.76±10.00	*α* =0.78
GSI	46.49±7.08	57.17±2.76	62.65±2.82	70.68±4.49	56.65±10.84	*α* =0.97
PSDI	48.53±9.14	55.25±5.84	60.73±4.68	70.12±4.91	56.65±10.77	
PST	46.47±6.88	56.94±4.00	62.24±4.53	69.53±5.94	56.29±10.79	

SOM, somatization; O-C, obsessive-compulsivity; I-S, interpersonal sensitivity; DEP, depression; ANX, anxiety; HOS, anger-hostility; PHOB, phobic anxiety; PAR, paranoid ideation; PSY, psychoticism; GSI, global severity index; PSDI, positive symptom distress index; and PST, positive symptom total.

### Reliability

Reliability ranged from Cronbach’s Alpha=0.73 (HOS) to Alpha=0.89 (O-C, DEP) regarding the scales, and the reliability of the Global Score GSI was Alpha=0.97 (GSI).

### Differences Between the Four Groups of Different Psychologically Distressed Patients

Sociodemographic data: There were no statistically significant differences between the four groups, except the distribution of the variable gender; the group with no psychological distress had 68.3% women, the mildly distressed group 80%, the remarkably distressed group 86.1%, and the severely distressed group 81.7% (*χ*^2^=21.24, *p*<0.0001).

PHQ-4: The variation of the depression and the anxiety score as well as of the sum score of the PHQ-4 corresponded to the four different psychologically distressed groups (see Table 3). The four groups differed remarkably with high effect sizes (ranged from *η*^2^=0.40 for the depression score to *η*^2^=0.49 for the anxiety score and *η*^2^=0.51 for the PHQ sum score), and *post hoc* testing resulted in six from six differences.

**Table 3:** Differences between the four different psychologically distressed groups regarding patient health questionnaire (PHQ) and questions about private conflicts, job stress, and pain (mean and SD).

Psychological distress	No	Mild	Remarkable	Severe	Sum	Stat.
	No two BSI-scales≥60	Two or more BSI-scales and/or GSI≥60 and <63	Two or more BSI-scales and/or GSI≥63 and <70	Two or more BSI-scales and/or GSI≥70		
	268 (41.9%)	85 (13.3%)	166 (26%)	120 (18.8%)	639	
PHQ-D	0.44±0.48	0.82±0.62	1.15±0.60	1.71±0.78	0.91±0.76	*F*=139 *p*<0.0001 *η*^2^=0.40 Six differences
PHQ-A	0.36±0.43	0.87±0.53	1.21±0.69	1.81±0.67	0.92±0.79	*F*=200.65 *p*<0.0001 *η*^2^=0.49 Six differences
PHQ-Sum	0.40±0.39	0.84±0.49	1.18±0.56	1.76±0.63	0.92±0.72	*F*=223.35 *p*<0.0001 *η*^2^=0.51 Six differences
Private conflicts	0.59±0.75	0.94±0.92	1.40±1.04	1.88±1.05	1.09±1.04	*F*=64.12 *p*<0.0001 *η*^2^=0.23 Six differences
Job stress	1.12±0.99	1.44±0.91	1.92±0.99	2.25±0.98	1.58±1.07	*F*=46.64 *p*<0.0001 *η*^2^=0.18 S vs. N, M, R R vs. N, M
Pain	1.18±0.86	1.45±0.83	1.56±0.92	1.87±0.87	1.44±0.91	*F*=18.70 *p*<0.0001 *η*^2^=0.08 S vs. N, M, R R vs. N

PHQ-D, PHQ scale depression, mean score of: “Little interest or pleasure in doing things,” “Feeling down, depressed, or hopeless.” PHQ-A, PHQ scale anxiety, mean score of: “Feeling nervous, anxious or on edge,” “Not being able to stop or control worrying.” PHQ-Sum, PHQ sum scale, mean score of four items. Private conflicts=“Are you currently suffering from particular family or private stresses or conflicts? Job stress=“Are you currently suffering from particular professional stress? (time pressure, overwork, conflicts, fear of job-loss, and dissatisfaction with work…). Pain=mean score of: “Strong or very strong physical pain,” “Physical pain prevented me from living a normal life (household, leisure time, work….” M, mild psychological distress; N, no psychological distress; R, remarkable psychological distress; and S, severe psychological distress.

Conflicts and pain: The four groups differed remarkably regarding private conflicts (family or private stresses or conflicts) with a high effect size (see Table 3); *post hoc* testing resulted in six from six differences. The differences between the four groups regarding job stress (time pressure, overwork, conflicts, fear of job loss, and dissatisfaction with work) were remarkable with a high effect size (see Table 3); *post hoc* testing resulted in five from six differences (groups “no” and “mild distressed” did not differ). The differences between the four groups regarding pain were statistically significant (see Table 3) on a medium level, and the *post hoc* tests resulted in four of six differences (group “mild” did not differ from “no” and “remarkable distress”).

## Discussion

The present study aimed to investigate a new routine for analyzing Brief Symptom Inventory profiles in chronic pain patients in order to recognize the need for prevention and to treat psychological distress as comorbidity in medically ill patients (Baumeister et al., 2011). Hereby, the focus was on early identification for prevention as well as intervention, which was investigated in a large orthopedic sample with possible higher prevalence.

Hereby, the BSI showed in high psychological distress values in 45%. Out of these, 13% showed mild, 26% remarkable, and 19% severe psychological distress. These results again show high psychological distress in somatically ill patients.

Furthermore, the differentiation into remarkable and severe can be underlined by significantly higher psychological symptoms in these groups (*post hoc* differences between all three groups). These patients showed higher symptoms of depression and anxiety. Hereby, a lot of private conflicts as well as high job stress were observed. A significant increase in anxious and depressive symptoms were present in the three groups. In addition, pain intensity differed significantly between these two groups, whereby the pain only differed significantly between the extreme groups of the low as well as the severe psychological distress group. Therefore, these significant differentiations of the three groups is clearly visible using different scales and therefore should lead to clinical implications.

The following clinical implications, especially for prevention and intervention, can be drawn based on these new cut-offs. For the group with mild psychological distress, a short diagnostic interview and, if required, a few more tests are necessary in order to allocate precisely to low-threshold outpatient group programs for general health promotion in the preventive sense. A retesting in 4weeks is recommended.

For the group with remarkable psychological distress, the patients should be in-depth exploration at both the interview and the test and questionnaire level, from which specific interventions at the individual and/or group level might be deduced, most of which should be on an outpatient basis or, in individual cases, as inpatient psychological interventions. A close follow-up is indicated.

For the group with severe psychological distress, in-depth exploration (interviews, tests, and questionnaires) should lead to specific individual and/or group offers, which should ideally be inpatient; the follow-up must be tight. Hereby, it is important to offer a first long-term discussion in the subsequent days in the form of urgent interventions and/or crisis intervention.

In sum, the new tool for analyzing psychological distress based on the T-scores of the BSI resulted in four groups (no psychological distress, low psychological distress, remarkable psychological distress, and severe psychological distress). These four groups differ significantly in psychological symptoms such as pain, depression, and anxiety as well as showing more private conflicts and higher job stress. Therefore, the new evaluation strategy of the BSI will improve practice and research in prevention and intervention of orthopedic rehabilitation.

The strength of the study is the large sample of patients with psychological distress. However, limited is the generalizability of the results since exclusively patients with orthopedic disorders without information on their medication were investigated. Other diseases, such as diabetes mellitus, osteoporosis, and cardiac disorders, are also associated with psychological distress and were not present in the current sample either as comorbidities or as primary diagnoses. Hereby, it has to be noted that anxiety is a predictor for bone mineral density at the lumbar spine and femoral neck and, therefore, for fracture risk in postmenopausal women assessed for osteoporosis (Catalano et al., 2018). Postmenopausal women with depressive disorder have an elevated risk for osteoporosis (Atteritano et al., 2013). These patients should be investigated in future research validating the four categories with the clinical implications. In addition, the patients’ medication as well as objective measures were not assessed due to the anonymization of the data. However, selective serotonin reuptake inhibitors (SSRIs), first-line agents in the pharmacological treatment of mood and anxiety disorders, have been shown to affect bone metabolism as well as depression itself, negatively. Therefore, risk assessment and recommendations for prevention and treatment of bone disease in psychiatric patients should be considered (Fernandes et al., 2016).

It should be noted that the present results only refer to the self-assessment of patients with orthopedic problems or pain. Accordingly, the results cannot be transferred to other groups. The new evaluation routine should be used in further samples, e.g., psychiatric patients or students, to examine whether the results are generalizable.

## Data Availability Statement

The dataset used and analyzed in the current study is available from the corresponding author on reasonable request.

## Ethics Statement

The studies involving human participants were reviewed and approved by Deutsche Rentenversicherung Bund (AZ8021-6-8920). The patients/participants provided their written informed consent to participate in this study.

## Author Contributions

All authors listed have made a substantial, direct and intellectual contribution to the work, and approved it for publication.

## Conflict of Interest

The authors declare that the research was conducted in the absence of any commercial or financial relationships that could be construed as a potential conflict of interest.

## Publisher’s Note

All claims expressed in this article are solely those of the authors and do not necessarily represent those of their affiliated organizations, or those of the publisher, the editors and the reviewers. Any product that may be evaluated in this article, or claim that may be made by its manufacturer, is not guaranteed or endorsed by the publisher.
